# Identification of potential M2 macrophage-associated diagnostic biomarkers in coronary artery disease

**DOI:** 10.1042/BSR20221394

**Published:** 2022-12-12

**Authors:** Kunlin Li, Ruize Kong, Lijing Ma, Yu Cao, Wei Li, Rui Chen, Kunmei Gong, Lihong Jiang

**Affiliations:** 1 Yan’an Hospital Affiliated to Kunming Medical University, 245 Renmin East Road, Kunming City, Yunnan Province, China; 2 First People’s Hospital of Yunnan Province, 157 Jinbi Road, Kunming, Capital of Yunnan Province, China

**Keywords:** Coronary artery disease, Diagnosis, M2 macrophage, WGCNA

## Abstract

Background: M2 macrophages have been reported to be important in the progression of coronary artery disease (CAD). Thus, the present study aims at exploring the diagnostic value of M2 macrophage-associated genes in CAD.

Methods: Transcriptome profile of CAD and control samples were downloaded from Gene Expression Omnibus database. The proportion of immune cells was analyzed using cell type identification by estimating relative subsets of RNA transcripts. Weighted Gene Co-expression Network Analysis (WGCNA) was carried out to screen the relevant module associated with M2 macrophages. Differential CAD and control samples of expressed genes (DEGs) were identified by the limma R package. Functional enrichment analysis by means of the clusterProfiler R package. Least absolute shrinkage and selection operator (LASSO) and random forest (RF) algorithms were carried out to select signature genes. Receiver operating curves (ROC) were plotted to evaluate the diagnostic value of selected signature genes. The expressions of potential diagnostic markers were validated by RT-qPCR. The ceRNA network of diagnostic biomarkers was constructed via miRwalk and Starbase database. CMap database was used to screen candidate drugs in the treatment of CAD by targeting diagnostic biomarkers.

Results: A total of 166 M2 macrophage-associated genes were identified by WGCNA. By intersecting those genes with 879 DEGs, 53 M2 macrophage-associated DEGs were obtained in the present study. By LASSO, RF, and ROC analyses, C1orf105, CCL22, CRYGB, FRK, GAP43, REG1P, CALB1, and PTPN21 were identified as potential diagnostic biomarkers. RT-qPCR showed the consistent expression patterns of diagnostic biomarkers between GEO dataset and clinical samples. Perhexiline, alimemazine and mecamylamine were found to be potential drugs in the treatment of CAD.

Conclusion: We identified eight M2 macrophage-associated diagnostic biomarkers and candidate drugs for the CAD treatment.

## Introduction

As the population ages, the incidence of coronary artery disease (CAD) has increased, which is one of the most serious threats to human health and quality of life [1,2]. CAD is a heart condition caused by atherosclerosis (AS), a narrowing or blockage of the lumen of the coronary vessels, resulting in ischemia, hypoxia, or necrosis of the heart muscle [3]. According to the China Cardiovascular Health and Disease Report 2020, the mortality rate of coronary heart disease in China is 120.18 per 100,000 urban residents and 128.24 per 100,000 rural residents, seriously endangering the health of the population [4].

Coronary angiography (CAG) is the gold standard for detecting CAD [2], and the most common traditional biomarkers for the diagnosis of coronary heart disease including ultrasensitive C-reactive protein (hs-CRP) and creatine kinase MB (CK-MB) [5]. Recently discovered non-invasive biomarkers such as miRNA and metalloproteinase-1 (MMP1) can be used as new methods for the CAD prediction and diagnosis [6–8]. Traditional diagnostic tools such as CAG or biomarkers are rarely used for early diagnosis or the prognosis prediction of CAD. The acute myocardial infarction in CAD is a serious threat to the health of the population, and there is an urgent need to find new tools for the early diagnosis of CAD so that appropriate interventions can be made earlier and in a more timely manner.

Monocytes are associated with the progression of CAD and atherosclerosis. During the course of CAD, monocytes are recruited and activated into macrophages. Macrophages can form foam cells after uptake of oxidized low-density lipoprotein (oxLDL) via scavenger receptors [9–11]; they are stimulated by different factors to differentiate into M1 and M2 subtypes and play different roles in the course of CAD [12]. M1 macrophages can secrete proinflammatory factors and substances such as reactive oxygen species that can aggravate atherosclerosis [13]. Another type of macrophage is the alternatively activated macrophage (M2), which secretes anti-inflammatory factors such as IL-1 receptor agonists, IL-10 and collagen, producing anti-inflammatory effects [14]. It has been shown that large concentrations of M1 macrophages are found in the unstable portion of rupture-prone plaques, while large concentrations of M2 macrophages are found in stable atherosclerotic plaques [15].

In conclusion, M2 macrophages are linked to the development of CAD. Identification of M2 macrophages is critical for early detection and cause research of CAD. As a result, the goal of the present study is discovery genes related to M2 macrophages in CAD and examination of their diagnostic utility for CAD through bioinformatics.

## Materials and methods

### Data source

The mRNA expression profiles of whole blood cell from 87 CAD and 52 control samples were downloaded from the GSE20680 dataset. The mRNA expression data of whole blood cells from 11 CAD and 13 control samples were downloaded from the GSE42148 dataset. The lncRNA expression data in peripheral blood mononuclear cells of 93 CAD and 48 controls were sourced from GSE113079. In addition, the miRNA expression data of platelet in 12 CAD and 12 controls were obtained from the GSE28858 database.

### Identification of differentially expressed genes

GSE20680 and GSE42148 were merged to identify differentially expressed genes (DEGs) between CAD and control samples. To remove the batch effect between GSE20680 and GSE42148, the ComBat function in the sva package of R was applied. Principle component analysis (PCA) was performed to assess the distribution of data in GSE20680 and GSE42148. Limma R package was used to screen DEGs using adjusted *P*-value < 0.05 as criteria. Meanwhile, differentially expressed miRNAs (DEmiRNAs) in GSE28858 and differentially expressed lncRNAs (DElncRNAs) in GSE113079 between CAD and control samples were also identified by the limma R package with a *P*-value < 0.05.

### Distribution of immune cells

Cell type identification by estimating relative subsets of RNA transcripts (CIBERSORT) was used to analyze the landscape of CAD and control samples in the merged dataset. In the present study, we used the LM22 signature, which are markers of naive B cells, memory B cells, plasma cells, CD8^+^ T cells, CD4^+^ naive T cells, resting CD4^+^ memory T cells, activated CD4^+^ memory T cells, follicular helper T cells, regulatory T cells, γΔT cells, resting NK cells, activated NK cells, monocytes, M0 macrophages, M1 macrophages, M2 macrophages, resting dendritic cells, activated dendritic cells, resting mast cells, activated mast cells, eosinophils, and neutrophils. For each sample, the final CIBERSORT output estimates were normalized, to sum up to one and thus can be interpreted directly as cell fractions for comparison across different immune cell types and datasets.

### WGCNA analysis

We first constructed a sample clustering tree map to detect and eliminate outliers. Then, weighted gene co-expression network analysis (WGCNA) was performed based on the gene expressions and the proportions of M2 macrophages of CAD and control samples. The pick Soft Threshold function of WGCNA was used to calculate β from 1 to 20 in order to select the best soft threshold. Based on the selected soft threshold, the adjacency matrix was converted to a topological overlap matrix to construct the network, and the gene dendrogram and module color were established by using the degree of dissimilarity. We further divided the initial module by dynamic tree cutting and merged similar modules. The Pearson’s correlation coefficient between the module eigengenes and the infiltration levels of M2 macrophages was calculated to find out the most relevant module. Genes in the module with gene significance (GS) > 0.5 and module membership (MM) > 0.8 were considered as modular genes and used for downstream analysis.

### Identification and functional analysis of M2 macrophage-related DEGs

M2 macrophage-related DEGs were obtained by intersecting DEGs with modular genes. The function of those genes was analyzed by the clusterProfiler R package. The Kyoto Encyclopedia of Genes and Genomes (KEGG) pathways and Gene ontology (GO) terms with a *P*-value < 0.05 and gene count ≥ 2 were considered as significantly enriched. The enrichment results were displayed in a bar chart generated by the enrichplot R package. The relationship among those genes were analyzed by constructing protein–protein interaction (PPI) network via the STRING database (confidence = 0.4).

### Construction of diagnostic model by LASSO and RF

Thereafter, random forest (RF) and least absolute shrinkage and selection operator (LASSO) were applied to construct the diagnostic model. Briefly, for LASSO, all samples in the merged dataset were randomly divided into training and testing sets at a ratio of 7:3. Then, we applied the LASSO algorithm by using the glmnet package in R software, screened the gene signatures under the optimal lambda with the smallest classification error, and constructed the diagnostic model based on the gene signatures in the training set. Receiver operating curves (ROC), composed of sensitivity, specificity, and area under the curve (AUC), were performed in both training and testing sets to evaluate the performance of the LASSO diagnostic models. RF was performed by the caret R package, and gene features and their contributions were then analyzed by explaining the function in the DALEX R package. Also, the ROC curve was plotted to assess the performance of the RF model in the diagnosis of CAD.

### Identification of hub genes in CAD

Hub genes in CAD were obtained by overlapping gene signatures identified in LASSO and RF. The diagnostic value of hub genes was assessed by ROC curves. Hub genes with an AUC > 0.7 were considered as candidate diagnostic biomarkers in CAD. Subsequently, the miRwalk database was used to predict DEmiRNAs targeting diagnostic biomarkers, and the Starbase database was used to predict DElncRNAs interacting with DEmiRNAs. By integrating miRNA-diagnostic biomarker and lncRNA-miRNA relation pairs, a lncRNA-miRNA-diagnostic biomarker network was constructed and visualized by Cytoscape software. In addition, the Cmap database was applied to identify potential drugs targeting diagnostic biomarkers. In the study, diagnostic genes with up-regulated and down-regulated trends were loaded into the ‘QUICK QUERY’ page and selected with criteria of enrichment score ≥ 0.5 and *P*-value <0.05. Next, the 3D structure and physicochemical properties of potential drugs were downloaded from the PubChem and DrugBank databases.

### RT-qPCR

Total RNA of blood samples from CAD (*N*=11) and controls (*N*=11) were extracted by TRIzol Reagent (Life Technologies, CA, U.S.A.). After detecting the concentration and the purity of RNA, qualified RNA was used for reverse transcription using sweScript RT I First strabd cDNA SynthesisAll-in-OneTM First-Strand cDNA Synthesis Kit (Servicebio, Wuhan, China). Then qPCR was performed using 2xUniversal Blue SYBR Green qPCR Master Mix (Servicebio, Wuhan, China) under the thermal cycling conditions: 40 cycles at 95°C for 60 s, 95°C for 20 s, 55°C for 20 s, and 72°C for the 30 s. The 2^−△△Ct^ method was used to calculate gene expressions. The primer sequences used in the present study were given in Table 1.

**Table 1:** Primers for qPCR used in the present study

Gene	Sequence (5′ to 3′)
C1orf105	F: AATGCCCTCATCCCTTCTCC
	R: TCACTGCCTCGTTGTCTTGC
CALB1	F: GTCATCCCTCATCACAGCC
	R: TCCAGGTAACCACTTCCGT
CCL22	F: CGCTTCAAGCAACTGAGGCA
	R: AGGGGCAGACGGTAACGGAC
CRYGB	F: CGCTCCTGCTGCCTCATCCC
	R: CCCCCCAATCAAGAAACCTC
FRK	F: CTTTGCTCTCCCCAGTCAC
	R: ATCCCCCTTCATCCAGTCT
GAP43	F: CGCACTCCACTTTTTACCTT
	R: CATCATTTTTTTCAACCCTT
REG1P	F: GGAAGGTAATAGGTGCTCTG
	R: AGGATGTTTTTGAATGGGAT
PTPN21	F: CGGATTTTGGTGACTTTGAT
	R: CACTTTTTGGGTTGCTTCTT
GAPDH	F: CCCATCACCATCTTCCAGG
	R: CATCACGCCACAGTTTCCC

## Results

### Identification of DEGs between CAD and controls

**Figure 1:**
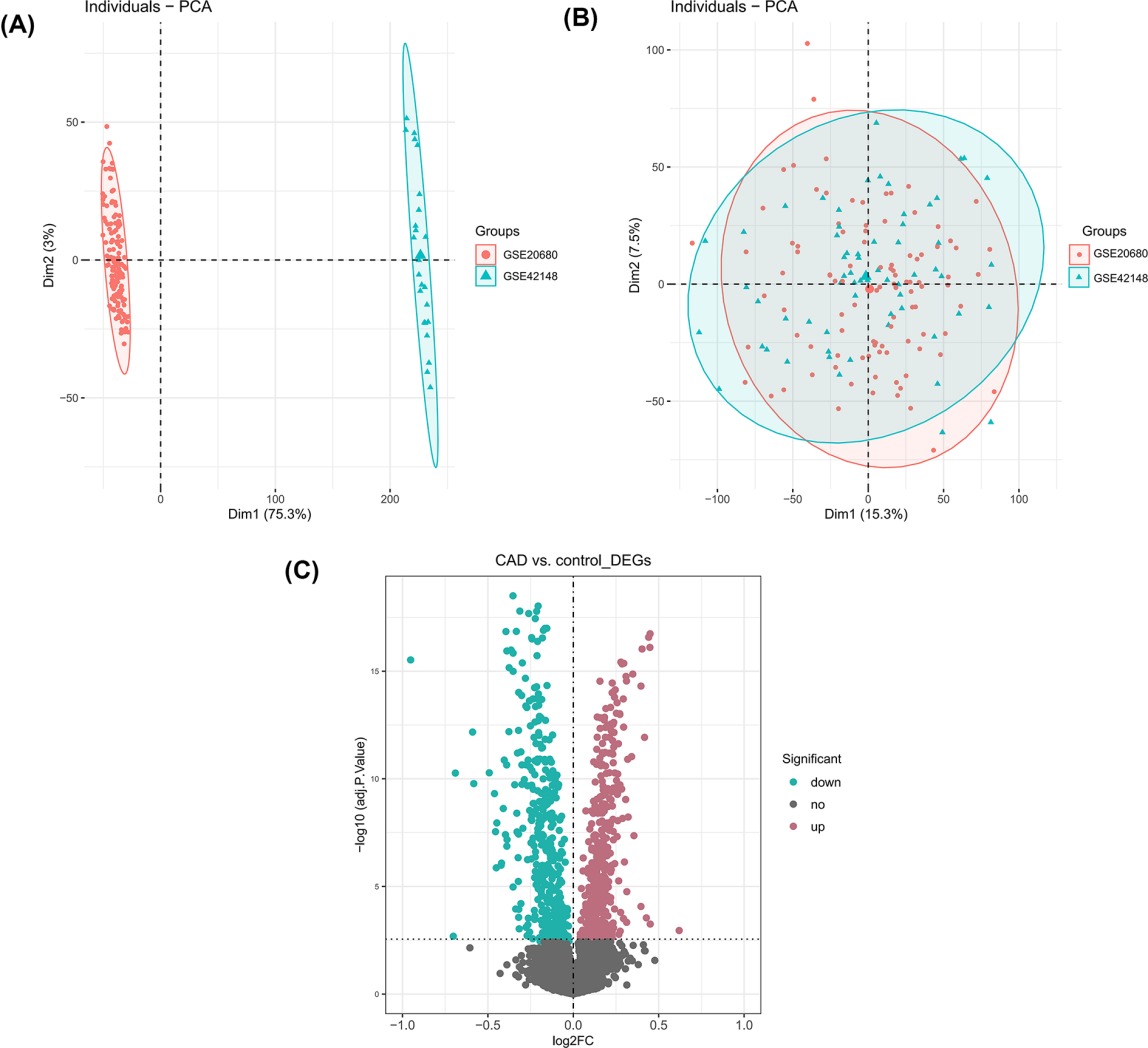
Identification of DEGs between CAD and control samples (**A**) PCA before removing batch effect, red dots represent GSE20680 data, and azure dots represent GSE42148 data. (**B**) PCA after removing batch effect, red dots represent GSE20680 data, and azure dots represent GSE42148 data. (**C**) Volcano plot of the merged dataset. Turquoise green ones represented down-regulation, dark reddish purple ones indicated up-regulation, and gray ones were the rest of the DEGs. The dashed horizontal and vertical axes indicate the logFC absolute threshold of 1 and the *P*-value threshold of 0.05, respectively.

The whole flowchart of the study procedure is shown in Supplementary Figure S1. PCA analysis showed that the data in GSE20680 and GSE42148 were distributed in distinct clusters (Figure 1A). After removing the batch effect (Figure 1B), we merged GSE20680 and GSE42148 to identify DEGs between CAD and controls. A total of 879 DEGs, including 446 up-regulated and 433 down-regulated genes, were identified (Supplementary Table S1 and Figure 1C).

### Identification of the key module associated with M2 macrophages

**Figure 2:**
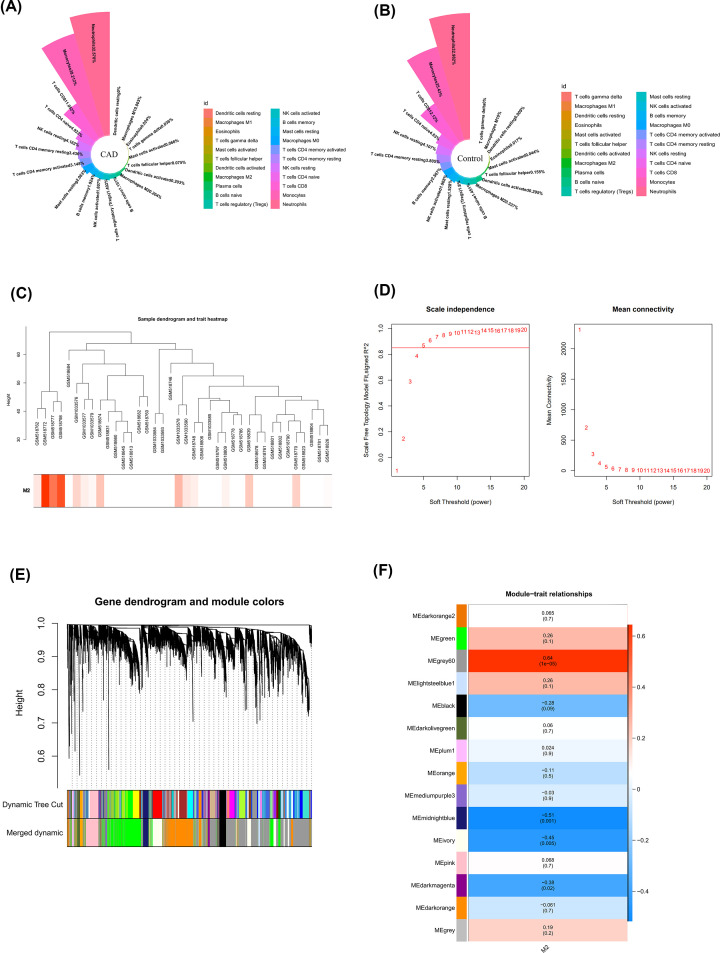
Identification of DEGs between CAD and control samples (**A,B**) Nightingale rose diagram of the proportion of immune cells in the CAD and normal group samples. Each color in the graph represents one immune cell and the larger the area of each colour, the greater the proportion of cells represented. (**C**) Construction of sample clusters and phenotypic trait heat maps based on M2 data from CAD and normal samples. (**D**) Determine soft thresholds. optimal soft threshold power was 5, in which *R*^2^ was approximately 0.85. (**E**) Cluster dendrogram of co-expressed genes in CAD. (**F**) Module–trait relationships in M2 macrophages. Each cell contains the corresponding correlation and *P*-value.

Next, we used CIBERSORT to investigate the distribution of immune cells in CAD and control samples (Supplementary Table S2). It can be found that the top 5 immune cells in CAD samples were resting memory CD4 T cells, resting NK cells, naïve CD4 T cells, monocytes, and neutrophils (Figure 2A), and the top 5 immune cells in control samples were resting NK cells, naïve CD4 T cells, CD8 T cells, monocytes, and neutrophils (Figure 2B). To find the genes associated with M2 macrophages, we performed WGCNA. According to the sample clustering result, no outlier samples were detected, and the sample dendrogram and trait heatmap were built as shown in Figure 2C. By using the pick Soft Threshold function of WGCNA, the optimal soft threshold power was determined as 5, in which *R*^2^ was approximately 0.85 (Figure 2D). Finally, after merging similar modules, 15 modules were identified from the co-expression network (Figure 2E). According to the module–trait relationships in Figure 2F, the grey60 module was most relevant to M2 macrophages (cor = 0.64, *P*-value < 0.01).

### Identification of M2 macrophages-related DEGs in CAD

**Figure 3:**
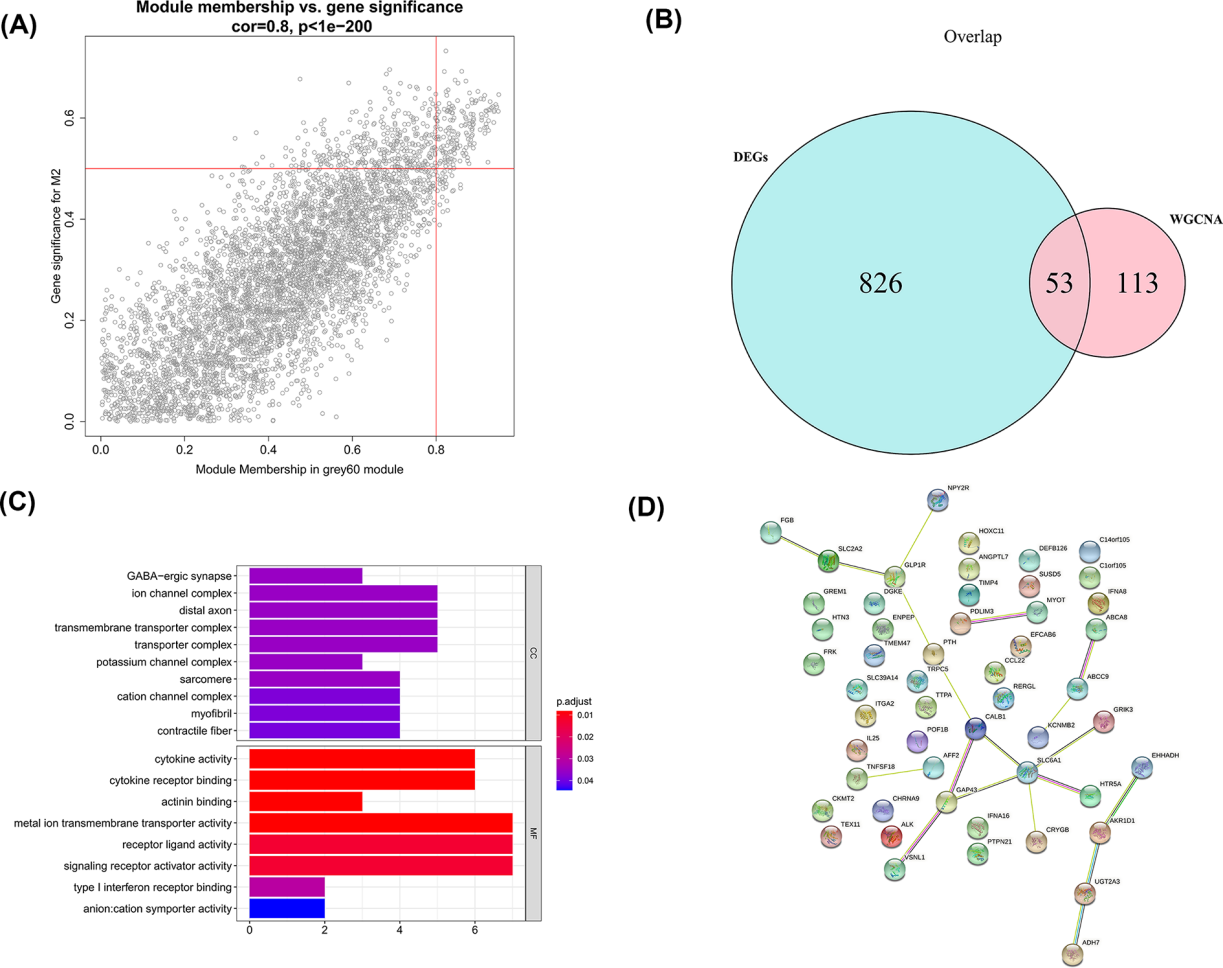
Identification of M2 macrophages-related DEGs in CAD (**A**) Scatter plot of module eigengenes in the grey60 module. (**B**) Screening for M2 macrophages-related DEGs. Blue represents genes differentially expressed between CAD/Control, and pink represents WGCNA acquisition of M2-related key module genes. (**C**) Bar chart of GO enrichment for M2 macrophages-related DEGs. The vertical coordinate indicates the enriched GO Term, the bar length indicates the number of key module genes enriched by that GO Term, and the color from blue to red indicates the low to high confidence level of the results. (**D**) A network of protein interactions for M2 macrophages-related DEGs.

Using GS > 0.5 and MM > 0.8, 166 genes in the grey60 module were obtained (Figure 3A). Then by overlapping those genes with DEGs, 53 M2 macrophage-related DEGs were obtained (Figure 3B). The M2 macrophage-related DEGs were significantly enriched into 13 cellular components, including GABA-ergic synapse, ion channel complex, distal axon, transmembrane transporter complex, transporter complex, potassium channel complex, sarcomere, cation channel complex, myofibril, contractile fiber, integral component of postsynaptic membrane, intrinsic component of postsynaptic membrane, axon terminus, and eight molecular functions, including cytokine activity, cytokine receptor binding, actinin binding, metal ion transmembrane transporter activity, receptor ligand activity, signaling receptor activator activity, type I interferon receptor binding and anion: cation symporter activity (Figure 3C). Furthermore, some M2 macrophage-related DEGs had interactions with each other via the STRING database (Figure 3D).

### Identification of diagnostic biomarkers in CAD

**Figure 4:**
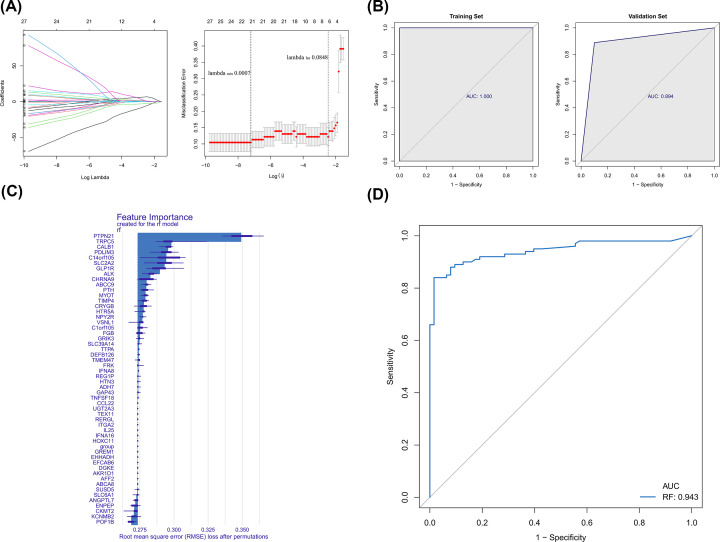
Identification of diagnostic signature for CAD by LASSO and RF algorithms (**A**) LASSO regression of the 53 M2 macrophage-related DEGs (left). Cross-validation for tuning the parameter selection in the LASSO regression (right). (**B**) Evaluation and validation of LASSO diagnostic model by ROC curves. (**C**) Importance of genetic variables in RF models. (**D**) Evaluation of RF diagnostic model by ROC curve.

**Figure 5:**
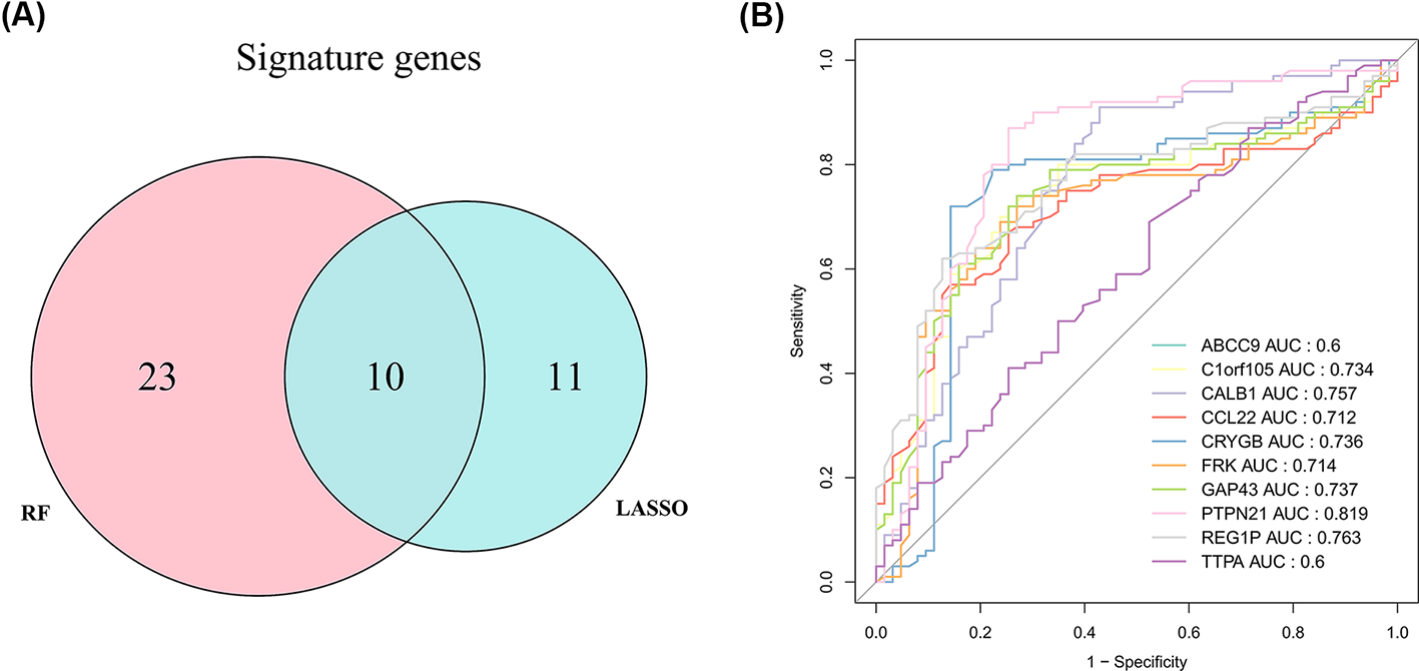
Identification of key diagnostic biomarkers for CAD (**A**) Intersection of LASSO diagnostic model genes with RF model genes. Blue represents LASSO diagnostic model genes; pink represents RF model genes. (**B**) ROC curves for candidate biomarkers.

**Figure 6:**
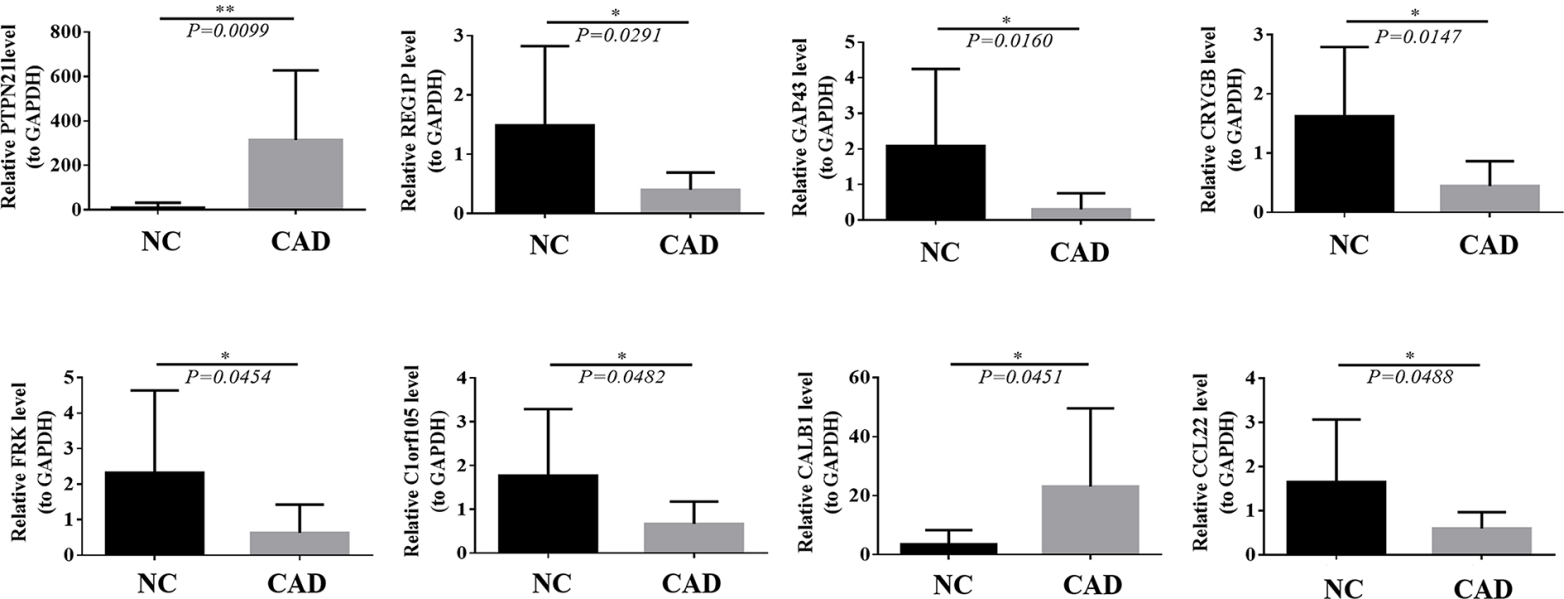
Detection of diagnostic biomarker expression by RT-qPCR in CAD and control samples. Data are expressed as mean ± SEM (n = 10), compared to control, * P < 0. 0 or ** P < 0. 01.

By LASSO, 21 gene signatures were identified (Figure 4A), including ABCC9, AFF2, C1orf105, CALB1, CCL22, CRYGB, DGKE, EFCAB6, EHHADH, FRK, GAP43, HOXC11, KCNMB2, POF1B, PTPN21, REG1P, RERGL, SUSD5, TEX11, TTPA, and UGT2A3. ROC showed that the LASSO model had good performance in both training (AUC = 1) and testing sets (AUC = 0.894) (Figure 4B). By RF algorithm, CCL22, TNFSF18, GAP43, ADH7, HTN3, REG1P, IFNA8, FRK, TMEM47, DEFB126, TTPA, SLC39A14, GRIK3, FGB, C1orf105, VSNL1, NPY2R, HTR5A, CRYGB, TIMP4, MYOT, PTH, ABCC9, CHRNA9, ALK, GLP1R, SLC2A2, C14orf105, PDLIM3, CALB1, TRPC5, and PTPN2 were selected as gene signatures (Figure 4C). The AUC value for the RF model was 0.943 (Figure 4D), indicating its ability in distinguishing CAD and control samples. By overlapping gene signatures in LASSO and RF models, 10 hub genes were acquired (Figure 5A). It can be seen from ROC curves that C1orf105, CALB1, CCL22, CRYGB, FRK, GAP43, PTPN21, and REG1P may act as the potential diagnostic biomarkers (Figure 5B). The expressions of PTPN21 and CALB1 were significantly elevated, while the expressions of C1orf105, CCL22, CRYGB, FRK, GAP43, and REG1P were significantly decreased in the CAD samples of the merged dataset (Supplementary Figure S2). Consensus expression patterns of diagnostic biomarkers were found in CAD and control samples by RT-qPCR (Figure 6).

### Identification of lncRNAs, miRNAs, and drugs targeting diagnostic biomarkers

**Figure 7:**
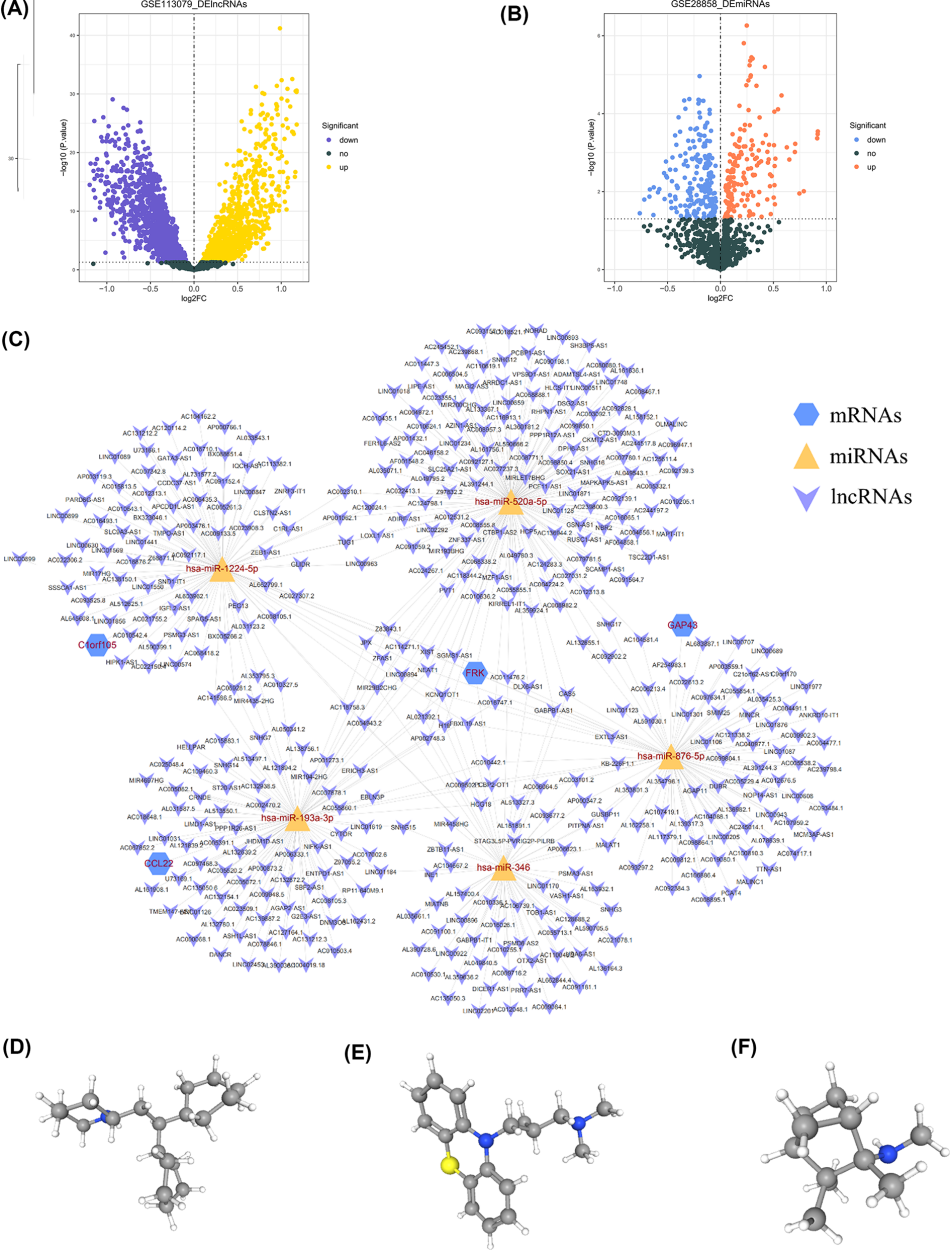
Identification of lncRNAs, miRNAs, drugs targeting diagnostic biomarkers (**A**) Volcano plot of differentially expressed lncRNAs in the GSE113079 dataset. Purple dot represents down-regulated expression, and yellow dot represents up-regulated expression. (**B**) Volcano plot of differentially expressed miRNAs in the GSE28858 dataset. Blue dot represents down-regulated expression, orange dot represents up-regulated expression. (**C**) The diagnostic biomarker based ceRNA regulatory network in CAD. Blue hexagons represent differentially expressed mRNAs, yellow inverted triangular cones represent lncRNAs, and purple triangles represent mRNAs. (**D–F**) Three-dimensional structures of potential therapeutic agents: perhexiline (PubChem CID: 4746 Molecular Formula: C19H35N), alimemazine (PubChem CID: 5574 Molecular Formula: C18H22N2S), and mecamylamine (PubChem CID: 4032 Molecular Formula: C11H21N)

A total of 2158 DElncRNAs, including 961 up-regulated and 1197 down-regulated lncRNAs, were identified in GSE113079 (Supplementary Table S3 and Figure 7A). A total of 369 DEmiRNAs, including 165 up-regulated and 204 down-regulated lncRNAs, were identified in GSE28858 (Supplementary Table S4 and Figure 7B). Moreover, 37 DEmiRNA-diagnostic biomarkers and 488 DElncRNA–DEmiRNA pairs were found by miRwalk and Starbase database. Finally, a lncRNA-miRNA-diagnostic biomarker network was constructed, which was composed of five miRNAs (mir-520a-5p, mir-876-5p, mir-346, mir-193a-3p, and mir-1224-5p) and four diagnostic biomarkers (C1orf105, CCL22, FRK, and GAP43) (Figure 7C). Besides, the 40 potential drugs targeting diagnostic biomarkers predicted in the treatment of CAD were summarized in Supplementary Table S5, the top 3 significantly identified drugs among others were perhexiline (PubChem CID: 4746, Figure 7D), alimemazine (PubChem CID: 5574, Figure 7E), and mecamylamine (PubChem CID: 4032, Figure 7F). The Log*P* values of which are 6.2, 4.71, and 2.7, respectively, and the other chemico-physical properties (molecular formula, solubility, p*K*a, etc.) were presented in Supplementary Table S6. The obtained compounds may achieve positive treatment outcomes of CAD.

## Discussion

CAD is one of the major cardiovascular diseases that seriously endanger human health. The pathological basis of CAD is atherosclerosis of the coronary vessels [4]. It has been demonstrated that a range of immune cells are relevant to the advancement of coronary artery disease, such as CD4 T cells [16], Treg T cells [17], neutrophils [18], and dendritic cells [19]. Furthermore, macrophage activation has been linked to coronary heart disease; the ratio of M1/M2 macrophages was found to be positively correlated with the severity of CAD [20,21]. We utilized bioinformatics analysis in this work to seek modular genes strongly related with M2 macrophages in CAD patients and analyze them appropriately to discover biomarkers and forecast prospective treatment drugs.

In the present study, M2 macrophage-associated DEGs were significantly enriched in 13 cellular components, and several of them involved in ion exchange inside and outside the cell (ion channel complex, potassium channel complex, cation channel complex). Macrophage ion exchange has several functions in the development of CAD inflammation [22–25]. For instance, macrophage pyroptosis is often seen as NLRP3 inflammasome activation, and one of the mechanisms of NLRP3 inflammasome activation is potassium ion crossing the cytoplasmic membrane [26].

Moreover, among the mainly enriched 13 cellular components, some of them were involved in cellular material transport (transmembrane transporter complex, transporter complex). The transport of substances by macrophages is also critical in the development of inflammation [27–29]. For example, members of the ABC transporter superfamily, ABCA1 and ABCG1, facilitate cholesterol efflux from foam cells to the extracellular cholesterol receptors Apolipoprotein AI (ApoA-I) and high density lipoprotein (HDL), respectively, restricting foam cell development and slowing the progression of atherosclerosis [30,31].

In turn, the eight enriched molecular functions also contain multiple cytokine-related MFs (cytokine activity, cytokine receptor binding, receptor ligand activity, type I interferon receptor binding). Macrophages are polarized into a variety of phenotypes (e.g. M1/M2 type) based on their environment [32]. To counteract the sustained inflammatory response and tissue damage produced by M1 macrophages, the body induces M2 macrophage polarization by activating Th2 cells to release IL-4 and IL-13 and binding IL-4R [33]. Furthermore, IL-10 can control M2 macrophage activity by stimulating the downstream STAT3 signaling pathway [34]. M2 macrophages secrete anti-inflammatory factors that antagonize the chronic inflammatory response induced by M1 macrophages; in addition, M2 macrophages have a strong phagocytic capacity to remove debris and apoptotic cells and suppress inflammation [35]. On the other hand, M2 macrophages are prevalent in stable plaques. More importantly, the phenotypic changes between M1 and M2 macrophage types are reversible [36,37].

Subsequently, the ROC curve result illustrated, C1orf105, CALB1, CCL22, CRYGB, FRK, GAP43, PTPN21, and REG1P could be used as potential diagnostic biomarkers. Among these, CCL22, which is primarily produced by monocyte-derived alternative (M2) macrophages, is a member of the CC family of chemokines and is involved in monocyte movement and recruitment [38]. CCL22 is a marker of monocyte/macrophage migration characteristic of atherosclerotic lesions [39]. Harrison et al. reported that rs3768445 on chromosome 1q24.3 is located in a cluster of protein-coding genes (DNM3, PIGC, C1orf105) that are associated with vascular remodeling and abdominal aortic aneurysm risk [40]. Electroacupuncture (EA) therapy enhances cardiac function by inhibiting mRNA and protein expression of GAP43 in a study of myocardial infarction in coronary artery disease [41]. PTPN21 has been reported to enhance the resistance of PC12 cells to ischemia and hypoxia by activating cdk5 through the ERK1/2 signaling pathway [42]. In summary, the hub genes related with M2 macrophages that we searched include CCL22, C1orf105, GAP43, and PTPN21, all of which imply a relationship with cardiovascular disease, which is consistent with our findings.

We created a network comprising five miRNAs (mir-520a-5p, mir-876-5p, mir-346, mir-193a-3p, and mir-1224-5p) and four diagnostic biomarkers (C1orf105, CCL22, FRK, and GAP43). Shima et al. reported that miR-520 found in vascular stenosis was negatively correlated with COX-1 and PTGDS gene expression levels [43]. TGP inhibits MSU-induced inflammation in THP-1 macrophages by modulating the MALAT1/miR-876-5p/NLRP3 axis [44]. In addition, miR-346 controls SMC phenotypic transformation through interacting with the IFN- pathway [45]. It was found that TNFα down-regulates vitamin D receptor expression in a miR-346-dependent manner. As a result, it can promote the progression of cardiovascular disease [46]. Shen et al. mentioned that ERK regulates macrophage response to M1 and alters miR-193a-3p expression in exosomes via the NF-κB/JAK-STAT pathway [47].

Macrophages also play an important role in the multiple stages of coronary heart disease.The development of fibrosis after myocardial infarction is inevitable. Recent studies have shown that post-infarction fibrosis is closely associated with macrophages. Chowkwale et al. found that cardiac cells undergo both inflammatory and matrix remodeling processes through the secretion of cytokines, and that inflammation directly regulates TGFβ secretion through immune cell recruitment and indirectly through the up-regulation of macrophage phagocytosis [48]. In addition, macrophages promote cardiac recovery and remodelling by reducing fibrotic scarring [49–51]. circRNAs in M2 macrophage-derived extracellular vesicles (EVs) mediate the cardiac fibrosis process after myocardial infarction [52]. Taken together, this suggests that M2 macrophage polarization can effectively improve cardiac function and that M2 macrophage-associated genes can act as valid molecular markers for the diagnosis of patients with coronary artery disease.

To summarize, in the present study, we identified diagnostic markers associated with M2 macrophages for the first time in CAD. After analysis by the GSE20680 and GSE42148 datasets, we obtained 10 hub genes by overlapping the gene signatures in the LASSO and RF models, and by ROC curves, C1orf105, ALB1, CCL22, CRYGB, FRK, GAP43, PTPN21, and REG1P could be used as potential diagnostic biomarkers. Further, the RT-qPCR results of peripheral blood from our clinical collection of CAD patients showed the consistent expression patterns of diagnostic biomarkers. The hub genes we screened for, such as CCL22, were associated with M2 macrophages, which is consistent with our findings as well.

Nevertheless, there is still a few limitaions related to the study, first, only two sets of gene sample series (GSE20680 and GSE42148) were available through this study. More in-depth studies are needed with more samples of whole blood as well as coronary artery tissue regarding coronary artery disease. Second, there is a lack of definitive evidence for coregulation between non-coding RNAs and genes associated with M2 type macrophages. Further studies should be conducted to investigate functional validation experiments of M2 macrophage-associated non-coding RNAs in coronary heart disease.

In conclusion, the present study identified the diagnostic markers associated with M2-type macrophages in CAD, providing a corresponding biological target for clinical diagnosis to identify people at risk of coronary heart disease and for timely intervention, and providing a new idea for the prevention and treatment of coronary heart disease.

## Conclusion

We identified eight M2 macrophage-associated diagnostic biomarkers and candidate drugs for the CAD treatment.

## Supplementary Material

Supplementary Figures S1-S2

Supplementary Tables S1-S6

## Data Availability

The data used in this study were obtained from an online database. GSE20680 GSE42148, GSE113079 GSE113079, and GSE28858 data set from the GEO (https://www.ncbi.nlm.nih.gov/geo/). The ceRNA network of diagnostic biomarkers was constructed via miRwalk (http://mirwalk.umm.uni-heidelberg.de/) and Starbase (http://starbase.info/) database. CMap (https://cmap.ihmc.us/) database was used to screen candidate drugs in the treatment of CAD by targeting diagnostic biomarkers.

## Abbreviations

AS: atherosclerosis
AUC: area under the curve
CAD: coronary artery disease
CAG: coronary angiography
CC: cellular components
CK-MB: creatine kinase MB
DEG: differentially expressed gene
GEO: Gene Expression Omnibus
GO: Gene ontology
GS: gene significance
hs-CRP: ultrasensitive C-reactive protein
IL: interleukin
KEGG: the Kyoto Encyclopedia of Genes and Genomes
LASSO: least absolute shrinkage and selection operator
MF: molecular functions
MM: module membership
MMP1: metalloproteinase-1
oxLD: oxidized low-density lipoprotein
PCA: principle component analysis
PPI: protein–protein interaction
RF: random forest
ROC: receiver operating curves
RT-qPCR: real-time quantitative polymerase chain reaction
WGCNA: Weighted Gene Co-expression Network Analysis
