# Supplementary figures and images for: Identification of potential M2 macrophage-associated diagnostic biomarkers in coronary artery disease
Source: Biosci Rep. 2022 Dec 12;42(12):BSR20221394. doi: 10.1042/BSR20221394 (PMC9744721; doi:10.1042/BSR20221394)

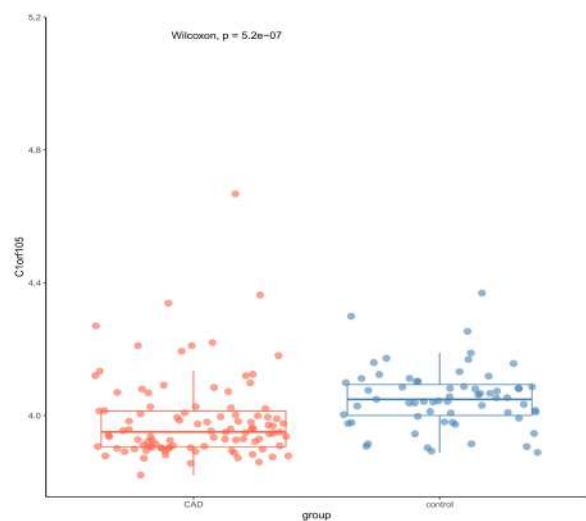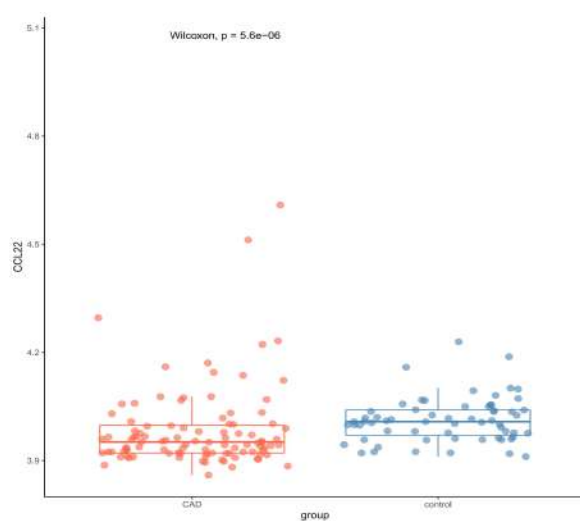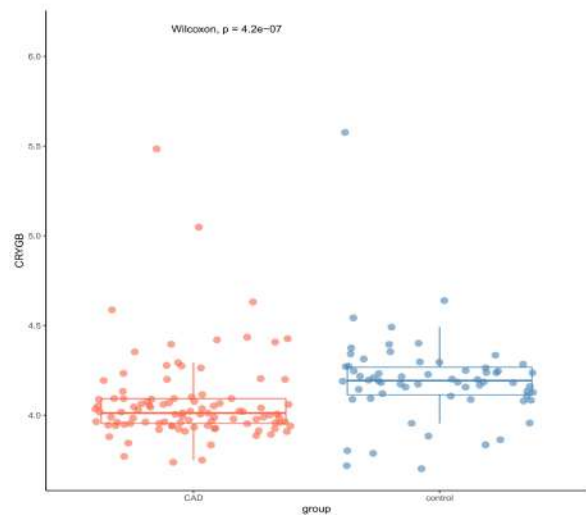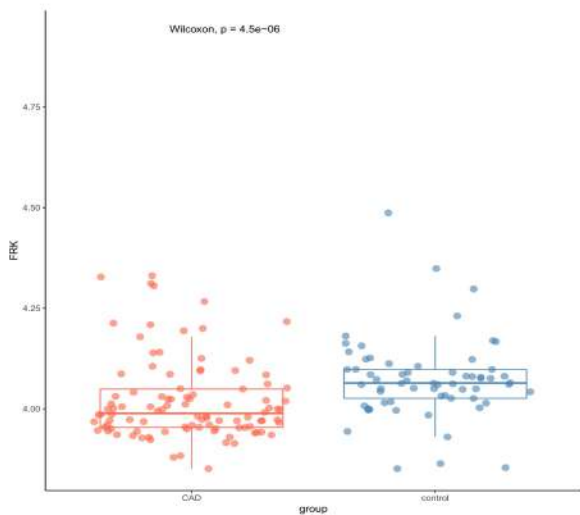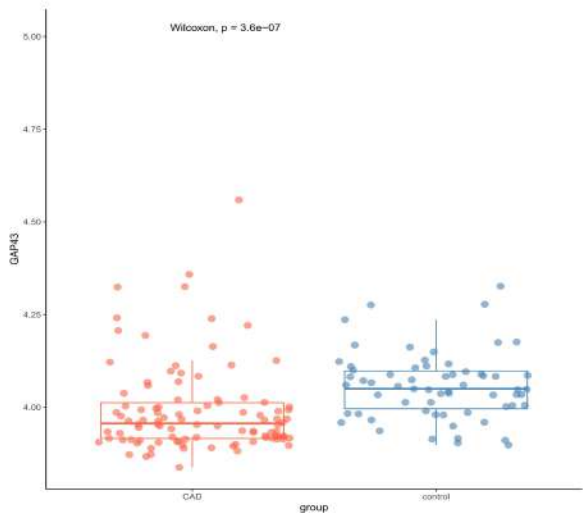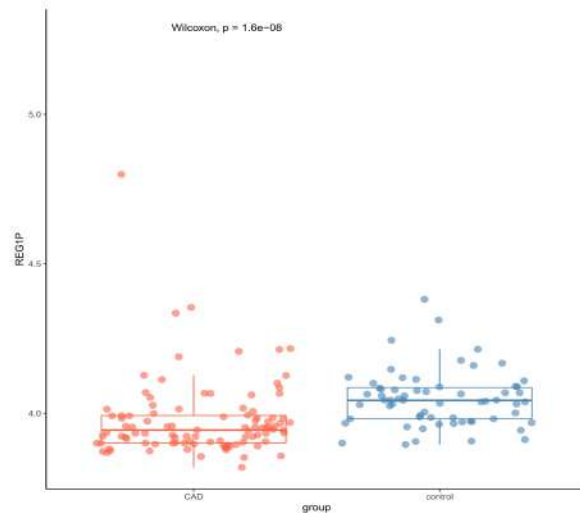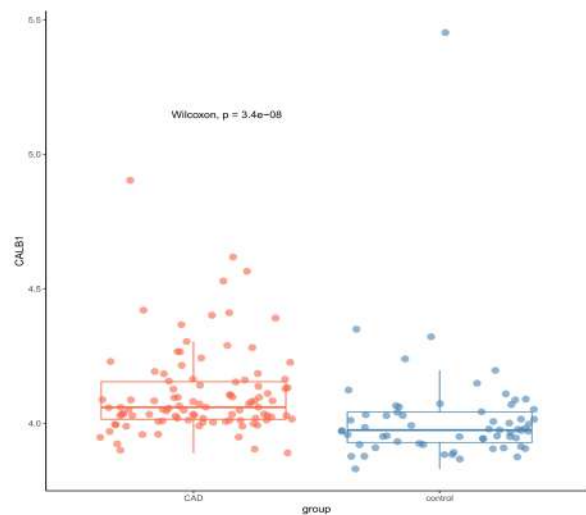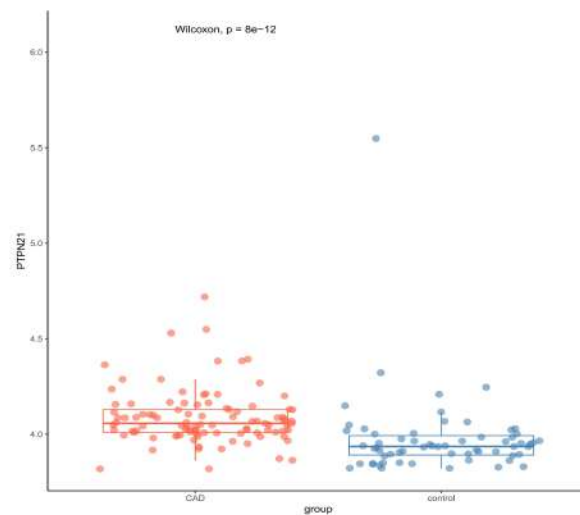

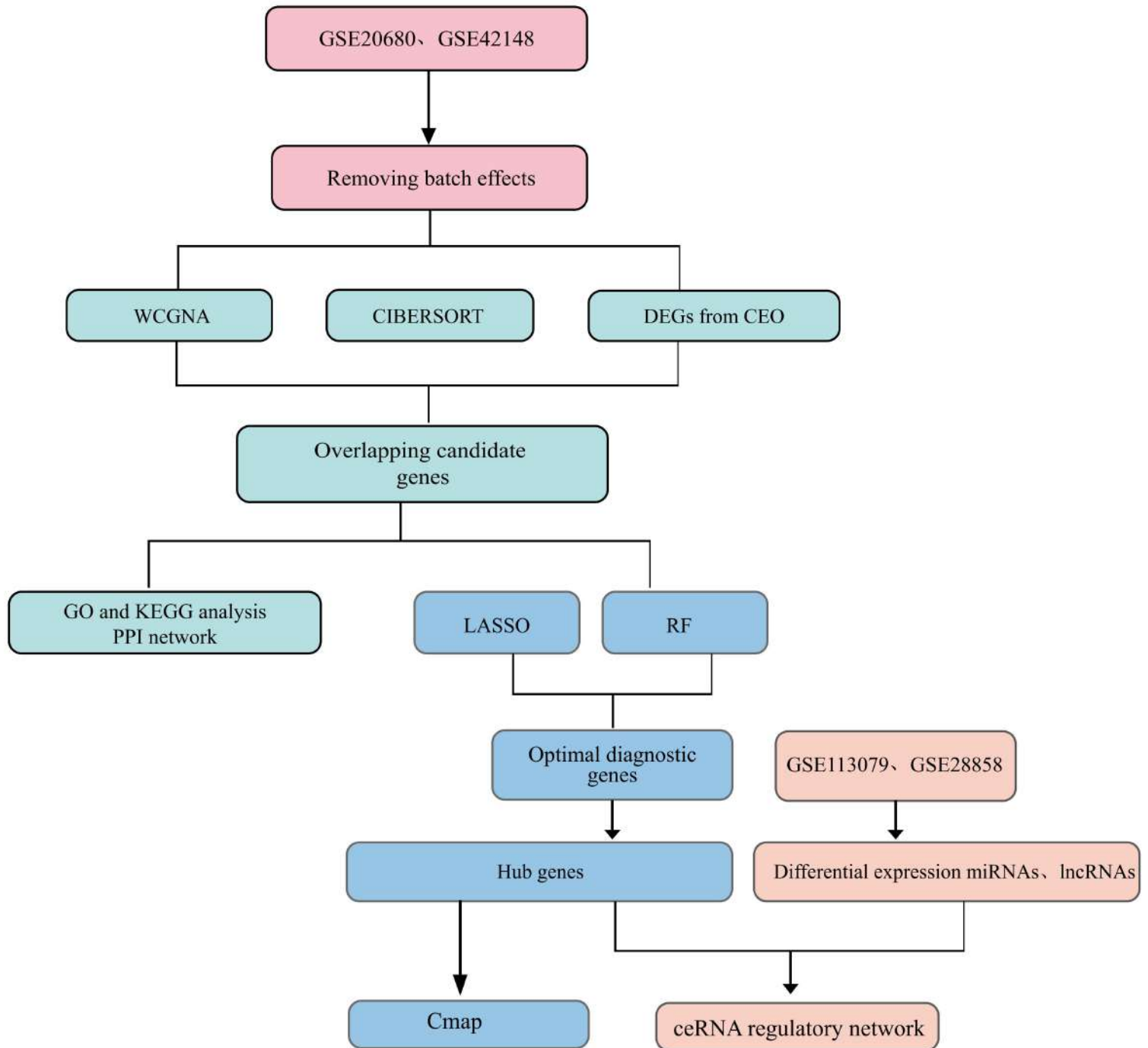

Supplement: Supplementary Figures S1-S2 [file BSR-2022-1394_supp.pdf]
